# Incorporating prior knowledge improves detection of differences in bacterial growth rate

**DOI:** 10.1186/s12918-015-0204-9

**Published:** 2015-09-21

**Authors:** Lydia M Rickett, Nick Pullen, Matthew Hartley, Cyril Zipfel, Sophien Kamoun, József Baranyi, Richard J. Morris

**Affiliations:** John Innes Centre, Norwich Research Park, Norwich, NR4 7UH UK; The Sainsbury Laboratory, Norwich Research Park, Colney Lane, Norwich, NR4 7RG UK; Institute of Food Research, Norwich Research Park, Colney Lane, Norwich, NR4 7UA UK

**Keywords:** Bayesian analysis, Nested sampling, Prior knowledge, Bacterial growth rate comparison

## Abstract

**Background:**

Robust statistical detection of differences in the bacterial growth rate can be challenging, particularly when dealing with small differences or noisy data. The Bayesian approach provides a consistent framework for inferring model parameters and comparing hypotheses. The method captures the full uncertainty of parameter values, whilst making effective use of prior knowledge about a given system to improve estimation.

**Results:**

We demonstrated the application of Bayesian analysis to bacterial growth curve comparison. Following extensive testing of the method, the analysis was applied to the large dataset of bacterial responses which are freely available at the web-resource, ComBase. Detection was found to be improved by using prior knowledge from clusters of previously analysed experimental results at similar environmental conditions. A comparison was also made to a more traditional statistical testing method, the *F*-test, and Bayesian analysis was found to perform more conclusively and to be capable of attributing significance to more subtle differences in growth rate.

**Conclusions:**

We have demonstrated that by making use of existing experimental knowledge, it is possible to significantly improve detection of differences in bacterial growth rate.

**Electronic supplementary material:**

The online version of this article (doi:10.1186/s12918-015-0204-9) contains supplementary material, which is available to authorized users.

## Background

This paper concentrates on a practical situation that occurs frequently in microbiology research. It is a widely accepted principle regarding bacterial batch cultures that when cells are inoculated into a growth-favouring environment, this determines a maximum specific growth rate [1]. It is a common task to measure this rate, often with a view to comparison across species or environmental conditions. However various issues can make such measurements difficult. The problems can be biological or technical. For example, the window of exponential phase could be too small to derive a statistically significant conclusion for the maximum specific growth rate. Also, differences in the rate of growth are often small, making the detection challenging, particularly when data is limited and noisy. In this paper we address these issues by taking advantage of existing experimental knowledge to improve detection.

**Fig. 1:**
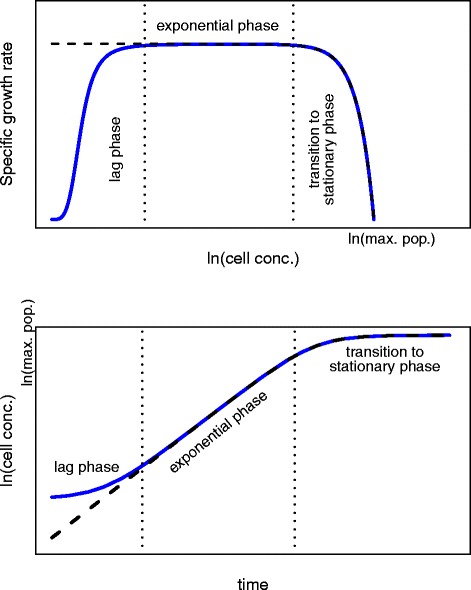
The model of Baranyi and Roberts incorporates three stages of bacterial growth. An illustration of the three stages of bacterial growth (lag, exponential and stationary) described in the model of Baranyi and Roberts [2, 3] (solid blue line). Other classical models for the population size, *x*(*t*), incorporate only the exponential and stationary phases (black dashed line) on the logarithmic scale. The top panel shows how the specific growth rate changes with the logarithm of the cell concentration during the three stages, whilst the bottom panel demonstrates how these phases correspond to the familiar sigmoidal shape that is observed when plotting the log cell concentration over time

The growth rate may be modulated by the state of the population: close to the inoculation, the cells are still adjusting to their new environment (lag phase), while at higher densities they slow down and eventually stop growing (transition to stationary phase). This behaviour is encapsulated in Fig. 1. During the lag phase, the specific growth rate is much less than its maximum and only a small amount of growth is detectable. To interpret growth measurements, a predictive mathematical model of bacterial growth can be used. Such models provide a means to describe the growth behaviour of a species over time by reducing the system to a set of fundamental parameters (such as the growth rate). These parameters must be estimated in order to fit the model to a given dataset. In this paper we use the model of Baranyi and Roberts [2, 3], which is able to describe all three stages of bacterial growth (lag, exponential and stationary). Some background on this model is given in Additional file 1. For this model, the widely used logarithm of the bacterial concentration is sigmoidal, so that a relatively linear phase is both preceded and followed by a transition phase characterised by small specific growth rates, as shown in Fig. 1. A number of classical models [4–6] for the population size, *x*(*t*), (satisfying the condition “ (*dx*/*dt*)/*x* is monotone decreasing” [7, 8]) have frequently been used for the logarithm of *x* too (for example see [9–11]). The justification is that, applied in this way, such models show the expected sigmoid pattern on the logarithmic scale, although applying them on this scale makes their use purely empirical. The techniques described in this paper, however, may easily be extended to such cases.

Established traditional statistical testing methods typically consist of an *F*-test or Akaike Information Criterion combined with a maximum likelihood optimisation approach which makes point estimates with the goal of finding the best fit to the data given the model or hypothesis. The (no longer supported) program MicroFit allowed for the statistical comparison of parameters between two bacterial growth data sets using an *F*-test. In the literature, one report used nonlinear regression techniques to fit the specific growth rates of strains of *Staphylococcus aureus* and utilised both a maximum-likelihood based *χ*^2^ test and principal-component analysis reference to compare restricted models (with one or more parameters assumed to be equal between strains) [12]. A separate study used linear spline regression to calculate growth slopes in measurements of different strains of *Escherichia coli* and termed curves as significantly different if they lay outside an estimated 95 % confidence interval [13].

Despite their frequent use in curve fitting problems, maximum likelihood and other optimisation techniques can often be misleading. A discussion of problems relating to optimisation methods can be found in [14]. One issue is the possibility of overfitting due to inadequate representation of measurement errors in the data [15], a problem that frequently leads to parameters being much more sharply defined than is justifiable given the data. Another issue is that the point estimate approach of optimisation ignores the contribution from the rest of parameter space and may miss alternative solutions [16]. Further, maximum likelihood approaches do not provide a framework to make use of prior knowledge to improve future estimation.

Using the Bayesian framework we may capture the full uncertainty of the problem, taking the whole of parameter space into account to make consistent predictions. We reduce the risk of overfitting since Bayesian techniques inherently account for the trade-off between model simplicity and goodness of fit [17]. Furthermore we may encapsulate existing knowledge through the prior probability and capitalise upon this knowledge to improve our inferences. In addition, the tools for model and hypothesis comparison are readily available through the calculation of the Bayes factor [16].

The success of Bayesian methods for parameter inference in biological systems has promoted an exciting new research area. A good review of this area can be found in [18]. Major developments in the application of Bayesian methods in general have been possible due to advances in sampling techniques. Nested sampling, as pioneered by Skilling offers a large improvement over multi-dimensional approaches such as highly computationally expensive Markov chain Monte Carlo methods, due to the reduction of the high-dimensional integrals that arise from Bayesian analysis to integrals over a single dimension [19, 20]. Recent applications include simulations of potential energy surfaces for protein folding [21], parameter inference for a model of circadian rhythms [22] and the analysis of experimental data for mice infected with *Salmonella enterica*, with relevance to alternative modelling techniques [23]. Also using nested sampling, a recent study considered how Bayesian analysis can address the problems associated with uncertainty when inferring parameters and comparing models for biological processes, particularly within the framework of experimental design [14].

In this paper we employ a nested sampling based Bayesian approach to infer parameters and compare bacterial growth curve data, in particular through comparison of the growth rate. Our work makes use of the ComBase database [24], which contains over 15,000 microbial growth curves collected under many different experimental conditions.

## Methods

### Model for bacterial growth

In this paper we use the 4 parameter model of Baranyi and Roberts [2, 3] which encompasses both the lag to exponential and exponential to stationary transitions of bacterial growth. Letting the bacterial concentration at time *t* be given by *x*(*t*), this model is described by

(1) $$ y(t) = \ln x(t) = y_{0} + \mu_{\max} A(t) - \ln\left(1+\frac{e^{\mu_{\max}A(t)}-1}{e^{(y_{\max}-y_{0})}}\right),   $$

(2) $$ A(t) = t - \frac{h_{0}}{\mu_{\max}} + \frac{\ln\left(1-e^{-\mu_{\max} t}+e^{-(\mu_{\max}t- h_{0})}\right)}{\mu_{\max}},   $$

where *y*_0_= ln*x*(0) and *y*_max_= ln*x*_max_, for *x*_max_ the maximum bacterial concentration. In addition *μ*_max_ denotes the maximum specific growth rate and *h*_0_=*λμ*_max_, where *λ* is the length of the lag phase. More details on the background of this model are available in Additional file 1. We note that although the bacterial concentration must be transformed to the ln*x* scale for use in the model, the concentration and parameter set are transformed afterwards to the more usual log10*x* scale in all of our results.

### Parameter inference using Bayesian analysis

Key to the task of parameter inference using Bayesian analysis is Bayes’ theorem, which encapsulates our inference about the parameter set, **p**, when using some hypothesis or model, *H*, given the observation of some data, **D**, and any background information, *I*, by calculation of the posterior probability distribution, *P*(**p**|**D**,*H*,*I*), where

(3) $$ P({\mathbf{p}}|{\mathbf{D}},H,I) = \frac{{P({\mathbf{D}}|{\mathbf{p}},H,I)P({\mathbf{p}}|H,I)}}{P({\mathbf{D}}|H,I)}.   $$

Here, *P*(**D**|**p**,*H*,*I*) (with shorthand $\mathcal {L}({\mathbf {p}})$) is the likelihood, *P*(**p**|*H*,*I*) (or *π*(**p**)) is the prior probability distribution without knowledge of the data and *P*(**D**|*H*,*I*) (or $\mathcal {Z}$) is known as the evidence and can be thought of as the probability of seeing the given data if hypothesis *H* is correct.

#### The likelihood function

Given a dataset with *N* independent data points with normally distributed errors, the appropriate log-likelihood function is

(4) $$ \log \mathcal{L} = -\sum_{i=1}^{N}\log\left(\sigma_{i}\sqrt{2\pi}\right)-\frac{1}{2}\sum_{i=1}^{N}\frac{(d_{i}-y_{i})^{2}}{{\sigma_{i}}^{2}},   $$

where *d*_*i*_ is the *y* component of the *i*-th data point and *y*_*i*_ denotes the *y* value obtained by applying the model using the *t* component of the *i*-th data point. The weighting or noise level is taken to be constant, so that *σ*_*i*_=*σ*. This assumption can be justified by considering that all data was collected using a viable count method, for which colonies resulting from serial dilutions of bacterial suspensions are counted and the results used to determine the bacterial concentration (number of colony forming units) in the original sample [24]. Since there is an optimum range of colony numbers for counting (between 50 and 300), one bacterial concentration that is 10 times bigger than another must be 10 times more diluted and the resulting error incurred by forward interpolation of the counted value will be 10 times greater. For this reason, we may assume that the errors in the measured bacterial concentration, *x*, are log-normally distributed, and therefore the errors in *y*= log10(*x*) are normally distributed. In our analysis *σ* may be prescribed (at *σ*_*F*_) or inferred as a parameter (*σ*_*I*_) using the Jeffreys prior [25]. In Additional file 1 we compare these two cases and show that it may be disadvantageous to prescribe *σ*. We recommend that unless we are sure of the noise level associated with our data, *σ* should be inferred.

Alternative measures may be used to include relevant data points for which the microbial concentration is too low to be detected by the given experimental method. Depending on the sensitivity of the method used when collecting the data, the threshold below which values were considered undetected was taken to be either 0.7 or 1.3 on the log10*x* scale. For these values, rather than using the log-likelihood function (Eq. 4), the probability was assigned by the uniform distribution between zero and the threshold, and zero above the threshold (although we include a small Gaussian tail in this region to avoid a singularity in the log likelihood). In this way, we may account for a lack of knowledge (equal likelihood) regarding the position of these points below the threshold and an absolute certainty that these points do not lie above the threshold.

#### The prior

We start by assuming a uniform prior probability (with bounds that are taken to scale with the data), reflecting a lack of prior information for all parameters. In the case that we have prior knowledge available that we wish to take into account, we can use an appropriate prior distribution. In this paper we have used a Gaussian or Cauchy prior (with appropriate bounds) to capture existing knowledge of the growth rate parameter. The informative Gaussian prior is the usual candidate for expressing definite prior information about a variable [26]. The weakly informative Cauchy distribution likewise encompasses a high probability region defined by prior information, but assigns more weight to values outside of this region, and so may be used when our prior information is less definite [27]. By making use of these three priors, we may decide whether to use clusters of pre-analysed growth curves as prior knowledge (by choosing an informative prior over the non-informative uniform prior) and to account for the strength of a given cluster and our confidence in its relevance (by choosing either a weakly informative or informative prior). A more detailed comparison and discussion on the choice of prior is given in the results section.

#### Calculation of the evidence

In Eq. 3 the evidence is essentially a normalisation factor and can be obtained through marginalisation by integrating over the parameters,

(5) $$ \mathcal{Z} = \int_{\mathbf{p}} \mathcal{L}({\mathbf{p}}) \mathcal{\pi}({\mathbf p}) \mathrm{d}\mathbf{p}.   $$

This integral may be obtained using nested sampling. Following references [19] and [20], we transform (5) into a one-dimensional integral over likelihood space. Denoting the elements of prior mass as d*X*=*π*(**p**)d**p**, we let *X*(*λ*) denote the proportion of the prior with likelihood greater than *λ*, so that

(6) $$ X(\lambda) = \int\limits_{\mathcal L({\mathbf{p}}) > \lambda} \pi({\mathbf{p}})\mathrm{d} {\mathbf{p}}.  $$

Using this terminology, we may re-write (5) as

(7) $$ \mathcal Z = {\int_{0}^{1}} \mathcal L(X) \mathrm{d} X,  $$

where $\mathcal L(X(\lambda)) \equiv \lambda $. The algorithm preserves an active set of *n* objects **p**_1_,…,**p**_*n*_ sampled across the prior. At each step the objects are sorted according to their calculated likelihood, the object with lowest likelihood (denoted $\mathcal L^{*}$) removed and a new sample point generated subject to the constraint $\mathcal L({\mathbf p}) > \mathcal {L}^{*}$. This process is repeated until termination, moving the objects up the likelihood gradient to regions of higher likelihood, even if these regions become disconnected in parameter space. For a detailed description of the choice of all control parameters used during the process, see Additional file 1.

Using the generated samples, the integral (7) can be approximated numerically by

(8) $$ \mathcal Z \approx \sum_{k=1}^{N} h_{k} \mathcal L_{k},  $$

where *h*_*k*_=*X*_*k*−1_−*X*_*k*_ is the width between successive sample points (and *X*_0_=1) and *N* is the total number of samples (the number of objects discarded from the active set plus those remaining in the active set at termination). Summary statistics of the posterior distribution are also readily available. For example given a parameter, *p*, with sequence of samples, *p*_*k*_, each with associated weight, $w_{k} = h_{k} \mathcal L_{k}/{\mathcal Z}$, the mean and standard deviation are given by

$${\fontsize{9.1}{12}{\begin{aligned} \text{mean}(p) = \sum_{k=1}^{N} w_{k} p_{k}, \quad \text{sd}(p) = {\left(\sum_{k=1}^{N} w_{k} {p_{k}}^{2} - \text{mean(p)}^{2}\right).}^{1/2} \end{aligned}}} $$

Markov chain Monte Carlo methods produce samples from parameter space that are equally weighted and hence can be used to gain an understanding of the underlying posterior distribution. The same is possible with nested sampling; staircase sampling can be used to generate a number of equally-weighted posterior samples [19]. We make use of this technique to explore the posterior trajectories of inferred bacterial growth curves following analysis.

### Model Comparison using Bayesian analysis

Given the evidence, Bayesian analysis also provides a framework for model comparison. Again making use of Bayes’ theorem, we can write the posterior probability of some model or hypothesis given the data as

(9) $$ P(H|{\mathbf{D}},I) = \frac{P({\mathbf{D}}|H,I)P(H|I)}{P({\mathbf{D}}|I)}.  $$

Given two such models or hypotheses, *H*_1_ and *H*_2_, to describe the same data, their respective posterior probabilities can be divided to obtain the ratio

(10) $$ \frac{P(H_{1}|{\mathbf{D}},I)}{P(H_{2}|{\mathbf{D}},I)} = \frac{P({\mathbf{D}}|H_{1},I)P(H_{1}|I)}{P({\mathbf{D}}|H_{2},I)P(H_{2}|I)},  $$

and assuming equal prior probabilities for the hypotheses (*P*(*H*_1_|*I*)=*P*(*H*_2_|*I*)),

(11) $$ \frac{P(H_{1}|{\mathbf{D}},I)}{P(H_{2}|{\mathbf{D}},I)} = \frac{P({\mathbf{D}}|H_{1},I)}{P({\mathbf{D}}|H_{2},I)} = \frac{\mathcal{Z}_{1}}{\mathcal{Z}_{2}} = \mathcal B_{12}.  $$

The ratio of evidences ${\mathcal {Z}_{1}}/{\mathcal {Z}_{2}}$ is known as the *Bayes factor* and provides a metric for model comparison [28]. Jeffreys’ scale [29] provides a useful qualitative tool for interpretation of this factor (by calculating $\ln (\mathcal B_{12})$), as shown in Table 1. The table shows the grading of decisiveness of evidence to support or reject the hypothesis *H*_2_. If the log-Bayes factor is negative it can trivially be reversed to provide evidence against the competing hypothesis. It should be noted that in contrast to null hypothesis significance testing (rejecting or failing to reject the null) the Bayes factor provides the ability to rank hypotheses.

**Table 1:** Jeffreys’ scale for interpreting the Bayes factor and rejecting hypothesis 2 compared to hypothesis 1

$2\ln \mathcal {B}_{12}$	Evidence against hypothesis 2 (*H*_2_)
0–2	Hardly worth mentioning
2–6	Has some substance
6–10	Strong
>10	Very strong

This model comparison framework is a useful and versatile tool. In Additional file 1 we consider the application to bacterial growth model comparison. In this paper, however we focus on the application to the comparison of growth rates for a single model.

#### Hypothesis testing to compare growth rates between two curves

As mentioned in the introduction, different bacterial strains or changes in the environment can result in different characteristic growth rates. Given two growth curves, therefore, it is often of great interest to determine whether the two growth rates are significantly different. We compute the evidence and posterior probability for each of three possible hypotheses to describe a pair of curves. In the first hypothesis (*H*_1_) the two curves are replicates and the same set of parameters can be used to describe each, in the second (*H*_2_) the two curves have the same growth rate but differ in all other parameters and in the third (*H*_3_) the curves share no common parameters. In all three cases both curves are fitted using the growth model of Eqs. 1 and (2) and the likelihood function is given by combining the individual likelihoods for the two curves (from Eq. 4). The individual hypotheses may then be compared using Bayes factor and the results interpreted using Jeffreys’ scale. This gives us a standardised scale of confidence in which to place results and allows us to consistently decide how many parameters are necessary to describe the two curves.

In order to compare to a traditional statistical testing method, we perform a similar analysis on the two datasets using an *F*-test. We test using two different models. In the *separated* model, optimisation is used to fit the growth model to the two datasets separately. In the *unified* model, optimisation is performed on the combined dataset such that the model is fitted independently for each dataset, but the same growth rate is used for both. The null hypothesis is given by the statement *the two curves have the same growth rate*. The *F*-test statistic is given by

(12) $$ F = \frac{\sum_{i=1}^{N_{1}+N_{2}}\left({y_{i}}^{u}-{y_{i}}^{s}\right)^{2}} {\sum_{i=1}^{N_{1}+N_{2}}\left(d_{i} - {y_{i}}^{s}\right)^{2}/ (N_{1}+N_{2}-1)},  $$

where the two datasets have *N*_1_ and *N*_2_ data points respectively, *d*_*i*_ is the *y* component of the *i*-th data point and *y*_*i*_^*s*^ and *y*_*i*_^*u*^ denote the *y* value obtained by applying the separated and unified models respectively using the *t* component of the *i*-th data point. Here the first degree of freedom is given by the difference between the number of parameters in the separated and combined models and the second degree of freedom by the difference between the total number of data points and the number of parameters in the combined model. The null distribution of the test statistic is the *f*-distribution and for each calculated statistic there is an associated probability density value. A commonly used threshold for the probability density value is 0.05 [30], below which the null hypothesis is rejected. In this case the difference in growth rate between the two growth curves is considered to be significant.

### Implementation

All results in the following sections were computed using our implementation of the above methods, *Bayesfit* and *Bayescompare*; the former may be used for inferring the parameters of the model for a single growth curve and the latter for the detection of differences in growth rate (via hypothesis testing). Both provide the means to incorporate prior knowledge as part of the analysis and allow for the use of a range of growth models (with different numbers of parameters). We have made these functions available as part of the R package *babar* (downloadable from CRAN [31]). Details and examples regarding the functionality of the package are available online as part of the CRAN documentation. Additional analysis and production of plots were performed using R (v. 3.2.1) [32] and additional packages, *ape* [33], *gridBase* [34], *corrplot* [35], *plyr* [36] and *ggplot2* [37].

## Results and discussion

### The growth rate is more precisely defined than the lag time associated parameter

Plots from our analysis show that the marginal posterior distribution is approximately unimodal for all parameters (Figure S10 in Additional file 1) and can be represented, for the sake of comparison, by the means and variances of posterior samples (Figure S11 in Additional file 1). Further, when using nested sampling to infer the parameters of a test curve, the correspondence with actual (known) parameter values is found to be good (especially for the growth rate), as is the accuracy of the inferred noise level, *σ*_*I*_ (Figure S12 in Additional file 1).

**Fig. 2:**
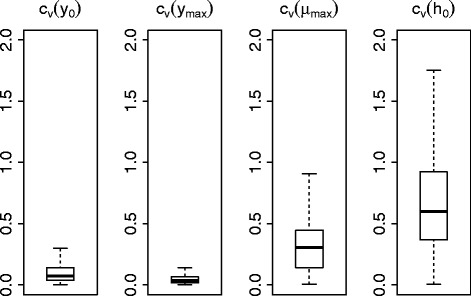
The inferred growth rate, *μ*_max_, is more precisely defined than the lag time associated *h*_0_. Boxplots for the coefficient of variation (*c*_*v*_= standard deviation/mean) for all parameters, calculated from the results of performing the Bayesian analysis using the 4 parameter model of Baranyi and Roberts over the entire database of growth curves from the ComBase database. Here the noise level, *σ*, is inferred for each curve. Whiskers show the minimum and maximum of the results in each case. Over the database, we find that the inferred growth rate, *μ*_max_, is the most precisely defined when comparing to *h*_0_=*λ* *μ*_max_ (where *λ* is the lag time)

Through calculation of the coefficient of variation for all parameters over the ComBase database, the growth parameter, *μ*_max_, is found to be more constrained by the data than the lag time associated parameter, *h*_0_ (see Fig. 2). This observation agrees with the work of others in the literature, where the growth rate has been found to be characteristic of the bacteria [1], whilst *h*_0_ has been found to be the least constrained by the data and therefore the most difficult to infer accurately [11]. Given that the minimum and maximum bacterial concentration parameters, *y*_0_ and *y*_max_, are largely influenced by experimental conditions (for instance, the inoculation level), this also lends evidence towards the hypothesis that the growth rate is the most important parameter for the purposes of meaningful curve comparison.

It must be noted, however, that the degree of accuracy of any parameter is subject to the quality of data. For example, we can expect that in the case that we have ample data based around the growth region, our inference of the growth rate will be more accurate than in the case that we only have sparse measurements. In the latter case, the increased uncertainty will naturally result in less tightly constrained behaviour in terms of the inferred growth dynamics. This kind of difference in behaviour is captured as part of our analysis and reflected in the inferred parameter variances (see Figure S13 in Additional file 1).

### The Bayes factor can be used to reliably detect differences in growth rate

**Fig. 3:**
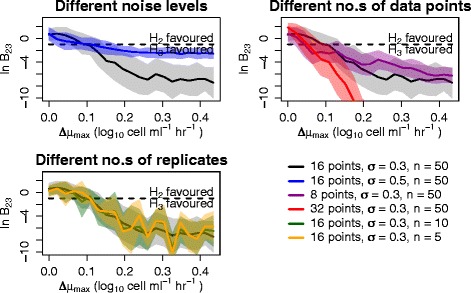
The difference in growth rate that can be detected is data-dependent. The results of comparing two curves and calculating the log Bayes factor, $\ln \mathcal {B}_{23}$, for hypothesis 2 (*H*_2_ following previous convention; *the curves have the same growth rate*) versus hypothesis 3 (*H*_3_ following previous convention; *the curves have different growth rates*) as the difference in growth rate, *Δ* *μ*_max_, between the two is increased. Here, the first curve is fixed, whilst the growth rate for the second is increased and *n* replicates with computationally random noise (with level *σ*) are generated for each difference in growth rate. The solid lines and shading represent the mean and standard deviation respectively for each case. The dashed line represents the line below which we favour hypothesis 3 over hypothesis 2 based on Jeffreys’ scale. The effect of using curves with different numbers of points and noise levels is explored, as well as using different numbers of replicates

The Bayes factor allows us to compare differences in the growth rate parameter, *μ*_max_. Figure 3 shows that the difference in growth rate that may be detected depends on the quality of the experimental data. Here a perturbation analysis is performed by comparing two curves as the difference in growth rate between the two increases. Following the methods definition of hypotheses 1 to 3, the evidence is obtainined for hypothesis 2 (*the curves have the same growth rate*) versus hypothesis 3 (*the curves have different growth rates*). During the analysis, two test curves (with pre-defined parameters) are generated, the first of which is fixed with growth rate 0.11, and the second of which has a growth rate which may be varied. The analysis is repeated *n* times for each difference in growth rate and the second curve is computationally re-generated for each repeat, so that the *n* resulting curves can be thought of as replicates, each with different random noise associated. The mean and standard deviation of the Bayes factor is calculated from the *n* repeats and this information, together with the known variance of the Bayes factor from Figure S2 in Additional file 1, can be used to illustrate the effect of lack of information on our analysis. We examine the effect of different noise levels, *σ*, datasets with different numbers of data points and different numbers of replicates. Unsurprisingly, the greater the amount and the better the quality of experimental data, the smaller the difference in growth rate that can be detected.

**Fig. 4:**
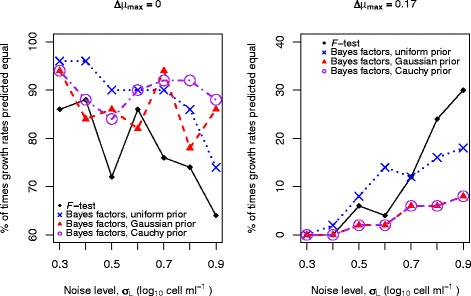
Bayes factor performs more consistently than an *F*-test when comparing growth rates. The results of using both an *F*-test (as described in the methods section) and Bayes factors (with the noise level, *σ*, inferred) on the same data to test whether two curves with 32 points have equal or different growth rates. We test two differences in growth rate, *Δ* *μ*_max_. The first curve is fixed, whilst for the second curve, 50 computationally random replicates are generated for each noise level *σ*_*L*_. For the Bayesian analysis, growth rates are considered to be different if there is substantial evidence for this (hypothesis 3) over equal growth rates (hypothesis 2). We also perform the analysis using prior knowledge (obtained from four previously analysed curves) for both curves. Here we make use of both the Gaussian and Cauchy priors and note that in the right hand plot the two are indistinguishable. Detection when using Bayes factors may be further improved by incorporating prior knowledge

Given the above findings, it is interesting to see how Bayesian analysis compares to traditional statistical testing methods under similar conditions. Figure 4 shows a comparison between the results of testing using the *F*-test described in the methods section and testing using Bayes factors. We consider the outcome of each testing method for two curves with and without the same growth rate at various different noise levels. Here, similarly to above, the first curve is fixed whilst the second is computationally regenerated 50 times for each noise level, resulting in 50 replicate curves each with different random noise. Initially, we consider the results when we use a uniform (uninformative) prior and find that Bayesian analysis produces more consistent results across the range of noise levels and correctly predicts equal growth rates more often than the *F*-test. The improvement in detection is more noticeable at larger noise levels (at times exceeding 10 %), since by using Bayesian analysis we may account for the noise level, *σ*. The same is true when the two growth rates are different; at larger noise levels there is a higher percentage of occurrences of correct detection when using Bayesian analysis with a uniform prior. For a few of the smaller noise levels, however, the *F*-test correctly predicts a difference in growth rate more often. Therefore, we next examine the effect of incorporating prior knowledge in our analysis to see if this provides a means to improve the overall level of detection.

### Prior knowledge can be used to improve detection of differences in growth rate

Bayes’ theorem provides a framework to make use of existing experimental knowledge through the use of the prior probability. In the following we evaluate whether making use of such knowledge can further improve our results with regards to growth rate comparison.

**Fig. 5:**
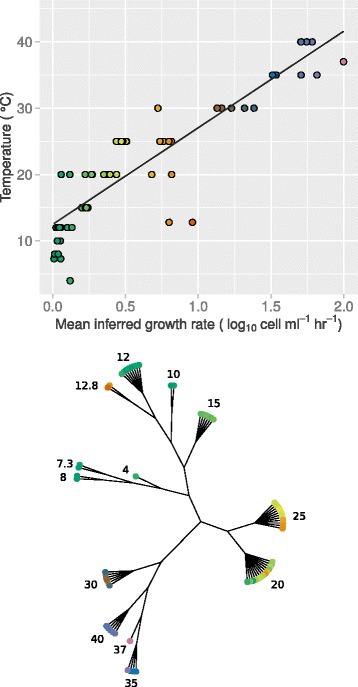
Correlations between temperature and growth rate may be used to build clusters of growth curves. (*Top*) Growth curves for the organism *Salmonella spp.* recorded at pH values of between 5 and 5.1 show a strong correlation (0.90) between their estimated growth rate and the temperature conditions in which they were grown. (*Bottom*) Clustering by temperature reveals clear grouping and when coloured by estimated growth rate this is well maintained. Clusters in the tree are labelled by temperature and the leaves coloured by growth rate. Colours are the same in both the top and bottom plots (high growth rate is purple, low growth rate is green). These clusters can therefore be used to give prior knowledge for improved parameter estimation

After running the Bayesian analysis over the large dataset of growth curves available at ComBase, an overall correlation of 0.63 was found between the recorded temperature and the estimated growth rate (Figure S14, Additional file 1). Due to the expected dependence of growth rate on bacterial species, it is reasonable to expect this correlation to be stronger when filtering by bacterial organism. Indeed, this is the case. For example, the 63 curves that were recorded at pH values of between 5 and 5.1 for the organism *Salmonella spp.* had a high correlation of 0.90 between temperature and growth rate (see top panel of Fig. 5).

We next investigate whether such a correlation may be used to build clusters of growth curves which could pre-inform priors for improved parameter estimation. The bottom panel of Fig. 5 shows the results of clustering the above-mentioned 63 curves by temperature, and colouring according to the inferred growth rate from our initial analysis. Here we use the R function, *hclust*, to perform average linkage hierarchical clustering, taking Euclidean distance as a metric. We see a good correspondence between the temperature clusters and their estimated growth rates, and similarly, when instead clustering by growth rate and colouring by temperature (Figure S15, Additional file 1). This suggests that such clusters could be exploited to inform the prior for the growth rate parameter. This in turn may lead to improved parameter estimation for a new growth curve under similar conditions.

Given justification in the methods section, we build informative Gaussian or Cauchy priors using the inferred means, *μ*_*i*_, and variances, *σ*_*i*_^2^, of growth rates from *N* previously analysed curves in a cluster. The overall mean and variance, *μ*_*cluster*_ and *σ*_*cluster*_^2^, for the growth rate are calculated using

(13) $$\begin{array}{*{20}l} \mu_{\text{cluster}} &= \sum_{i=1}^{N} \frac{\mu_{i}}{N},  \end{array} $$

(14) $$\begin{array}{*{20}l} {\sigma_{\text{cluster}}}^{2} &= \sum_{i=1}^{N} \frac{{\mu_{i}}^{2}}{N} - \left(\sum_{i=1}^{N} \frac{\mu_{i}}{N}\right)^{2} + \sum_{i=1}^{N} \frac{{\sigma_{i}}^{2}}{N},  \end{array} $$

where the variance is calculated using the law of total variance [38]. These values are then used in the prior (Figure S16 in Additional file 1 illustrates this method).

**Fig. 6:**
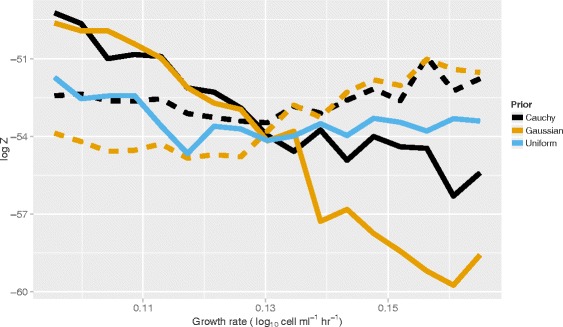
The highest ranked prior depends on closeness of growth rate to the growth rate cluster. Given two clusters of four simulated curves with parameters inferred by nested sampling, we plot the evidence, log*Z*, for analysing a new curve using a uniform (blue), Gaussian (orange) and Cauchy (black) prior as a function of position in-between the two clusters (in terms of growth rate). In the case of the Gaussian or Cauchy priors, the solid lines indicate that cluster A has been used for prior knowledge, whilst dashed lines indicate that cluster B has been used. Data sets from cluster A had growth rates between 0.09 and 0.11 and cluster B had rates between 0.15 and 0.17. Choosing a Gaussian prior is appropriate when the target growth curve is near to the cluster, but loses evidence as curves become more dissimilar from their cluster. The Cauchy prior can alleviate this to some extent, whilst the uniform prior has little change across growth rates

Figure 6 shows the results of using test data to illustrate the difference in evidence (and therefore ranking) between the three priors (uniform, Gaussian and Cauchy) as a function of the distance of the curve being analysed from the cluster used for prior knowledge. We illustrate this distance in terms of a difference in growth rate but, as shown above, this equates to a difference in temperature. Using a Gaussian or Cauchy prior, we observe a greater evidence as the curve becomes closer to the cluster. Since this results in a higher ranking for these choices of prior compared to the uniform prior, this suggests that either will improve parameter inference if the growth rate (or temperature) of the curve is close to that of the cluster. On the other hand, if we move too far away from the cluster, an informative prior may not be the most highly ranked choice, although the heavy-tailed Cauchy prior is more forgiving than the Gaussian. As expected, when no background knowledge informs the choice of prior (that is for a uniform prior) the evidence does not change significantly across the different data sets.

**Fig. 7:**
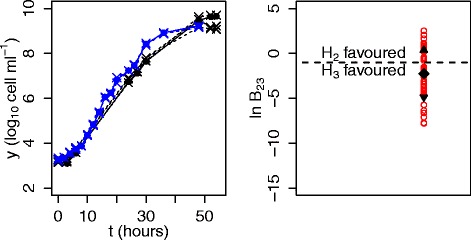
Using prior knowledge we can attribute significance to subtle differences in growth rate. We take *Escherichia coli* data from ComBase and use Bayes factors to compare the curve EcBook16_22_c (black solid line and filled circles in the left panel) with a curve with subtly different growth rate, EcGB_20_b (blue solid line and filled circles in the left panel), collected at a slightly different temperature and pH. The noise level, *σ*, is inferred and for each curve, we use prior knowledge from a cluster of previously analysed curves (dashed lines and crosses in the left panel), measured at the same temperature and pH. Growth rates are considered to be different if there is substantial evidence for this (hypothesis 3, *H*_3_) over equal growth rates (hypothesis 2, *H*_2_). Due to the variability in the stochastic algorithm, we compute the Bayes factor, *B*_23_, for 50 runs (red circles in the right panel) and compute the mean and standard deviation (black diamond and triangles in the right panel). Whilst the outcome of testing differences using an *F*-test is to accept the null hypotheses that the two curves have the same growth rate, a large proportion of runs for the Bayesian analysis result in the detection of a difference in growth rate

The improvement in parameter estimation when correctly using prior knowledge is found to be useful when comparing growth curves, as more precise estimation of parameters allows better detection of differences in growth rate. We return to the analysis of the previous section and Fig. 4 and find that when making appropriate use of clusters during hypothesis comparison, there is a general improvement in the percentage of occurrences of correct detection for a difference in growth rates. Due to this, the incorporation of prior knowledge results in a higher percentage of correct prediction than for the *F*-test in almost every case. As mentioned previously, this improvement is more noticeable for noisy data, where we may see up to a 25 % increase in correct prediction. When the growth rates are the same, it may be better to use a uniform prior. Since in practice we do not know how similar the growth rates are, these results suggest running the analysis both with and without prior knowledge and looking at the evidence value for the most appropriate prior choice in each case. In Fig. 7, we show an example of the application of these techniques to curves from the ComBase dataset. Here, we consider two clusters, each at a given temperature and pH value, and illustrate the subtle differences in growth rate that may be detected when comparing two curves, one from each cluster, using the rest of the curves as prior knowledge. In particular, whilst the difference between the two curves is too small to be deemed significant when using an *F*-test, for the Bayesian analysis a large proportion of the evidence supports the curves having different growth rates. This shows that we are able to attribute statistical significance to differences that are too small to be detected by an *F*-test.

These results are encouraging and point to the importance of methods, such as Bayesian analysis, which fully take into account both the quality of data and prior experimental knowledge when comparing growth rates between microbial response curves. Using Bayesian analysis we can improve on both the consistency and level of detection in our analysis, as well as answering the growing need to standardise statistical testing within a well-defined framework.

## Conclusions

Given the abundance of information available in many cases for bacterial response under differing environmental conditions, it is an appealing idea to use existing measurements to build a local model of environment space which may be used to address the challenging problem of detecting small differences in growth rate. We have shown that using Bayesian techniques we can consistently detect such differences. These techniques make use of the evidence and the Bayes factor as tools for ranking hypotheses. By taking into account prior knowledge through clusters of previous results under similar conditions and also by estimating the noise level of the data as part of the analysis, more subtle differences in growth rate may be shown to be significant. Such differences may be missed by traditional statistical testing methods such as an *F*-test.

When using clusters of results as prior information, however, we highlight that one should be careful that the given cluster is relevant to the curve to be analysed, so as not to obtain to misleading results. This should involve taking into account both how close the environmental conditions of the new curve are to that of the cluster and how tightly-defined the cluster itself is. A tightly-defined cluster that is a distance away from the new curve is likely to have a detrimental effect on the results. This is especially true when using the highly informative Gaussian prior. If it is suspected that this is the case, we recommend performing the analysis with both a uniform and Gaussian or Cauchy prior, in which case the evidence should be examined for the most appropriate choice. In some cases, it may be possible to combine clusters of results to form a more relevant but broader Gaussian or Cauchy prior.

We have also shown that the same framework can easily be used for model comparison (see Additional file 1). Indeed, in general, the evidence and Bayes factor are useful tools for conclusively testing hypotheses and models. We advocate the evidence as an alternative tool to traditional statistical testing methods and goodness of fit measurements in general.

There is scope for wider application of the techniques developed in this paper. The same methods may be used to analyse assays of bacterial growth in plants after, for example, spray inoculation. Here, it would be important to take into account lack of information since frequently only a small number of bacterial concentration measurements are collected. Optical density measurements also often lead to growth curves, which may be analysed by fitting to mathematical models such as those we have discussed [39, 40]. Although the present study has been restricted to bacterial growth curves, it is also possible to use the same techniques to analyse survival curves, for which there is a rich history of model development [41–43]. Further, the techniques presented in this paper can be applied outside of the field of microbial response entirely. Accurate measurement and comparison of the rate of some quantity is needed in many areas of chemistry and biology. For example, the study of enzyme kinetics requires completion of enzyme assays which measure, for instance, the change in substrate or product concentration over time in order to calculate a reaction rate. The many models available for mathematical analysis of population dynamics also lend themselves to analysis using the methods we have described. Another candidate for analysis is the estimation of real-time polymerase chain reaction curves using fluorescent reporters, for which the familiar phases of lag, growth and saturation can be observed [44]. In fact, many systems show a sigmoidal growth behaviour, introduced due to a combination of damping or disturbing factors that result in a levelling off of the rate for very small or large times, and could be analysed and compared using the models and techniques in this paper.

In the analysis of both bacterial growth curves and other systems showing similar growth behaviour, the growth rate is often the primary parameter of interest and other parameters may be thought of as noise since they are not strictly required as output. Here, it may make more sense to integrate out these “nuisance parameters” prior to the analysis [45]. Not only would this focus all efforts on accurate estimation of the rate, but, theoretically, it should also speed up the computation time. However, given the structure of models such as that defined by Eqs. 1 and 2, calculation of the likelihood function is likely to require numerical integration, which may in fact slow down the computation. It would be interesting, nevertheless, to determine whether such techniques could further improve detection of differences in growth rate.

## Availability of supporting data

The ComBase dataset is publicly available from http://www.combase.cc/.

## Additional file

Additional file 1
**Contains: material on our choice of control parameters in the analysis, an illustration of performing model comparison using the same framework and supplementary figures and tables.** (PDF 4639 Kb)
