# Supplementary material for: Incorporating prior knowledge improves detection of differences in bacterial growth rate
Source: BMC Syst Biol. 2015 Sep 21;9:60. doi: 10.1186/s12918-015-0204-9 (PMC4578766; doi:10.1186/s12918-015-0204-9)
Supplement: Additional file 1 — Contains: material on our choice of control parameters in the analysis, an illustration of performing model comparison using the same framework and supplementary figures and tables. (PDF 4639 Kb) [file 12918_2015_204_MOESM1_ESM.pdf]

Bayesian analysis and prior knowledge improve detection of differences in growth rate
Additional File 1: Supplementary material

Lydia M Rickett[1,2], Nick Pullen[1], Matthew Hartley[1], Cyril Zipfel[2], Sophien Kamoun[2], József Baranyi[3], and Richard J Morris[1]

[1] *John Innes Centre*
[2] *The Sainsbury Laboratory*
[3] *Institute of Food Research*

Contents

1 Background on the model of Baranyi and Roberts for bacterial growth **1**

2 Prescribing versus inferring the noise level, σ **2**

3 An explanation of the choice of control parameters used in the analysis **3**

4 Model comparison using the *Bayesfit* function **7**
4.1 A set of models for bacterial growth 7
4.2 Model comparison for comparing bacterial growth models 8

5 Supplementary figures **12**

1 Background on the model of Baranyi and Roberts for bacterial growth

A classical family of one-variable growth models states that in a constant physical environment, the expected number of individuals, $x(t)$, in the population at time t, can be described by the first order autonomous differential equation

$$\frac{dx}{dt} = \dot{x} = \mu(x)x, \tag{1}$$

with the initial value $x(0) = x_0$ and maximum population density, $x_{\max}$. Here, $\mu(x)$ is the so-called specific rate, which depends on the population size and has the following properties (see [1, 2]):

$$\mu(x) : [0, x_{\max}] \to \Re, \tag{2}$$

$$\mu(x) \text{ is continuously differentiable on } (0, x_{\max}), \tag{3}$$

$$\mu(x_0) > 0 \text{ and } \mu(x_{\max}) = 0, \tag{4}$$

$$\frac{d\mu(x)}{dx} \text{ is strictly negative on } (0, x_{\max}). \tag{5}$$

The last two conditions express that (i) at the initial conditions, however small the population is, it grows at a positive rate and (ii) the larger the population size, the smaller the specific rate, until the population size becomes $x_{\max}$, when growth stops. Under these conditions the above differential equation has a unique solution. This solution is monotone increasing and converges to $x_{\max}$ as $t \to \infty$.

For our practical task, it is more useful to study the logarithm of the population size and henceforth our main time-dependent variable is $y = \ln x(t)$. Accordingly, we use the notation $y_0 = \ln x_0$ and $y_{\max} = \ln x_{\max}$. With the above substitution, the specific growth rate becomes the slope of the $y(t)$ growth curve, and our model is:

$$\frac{dy}{dt} = \dot{y} = \mu(e^y), \quad y(0) = y_0. \tag{6}$$

For a bacterial population, it is assumed that, at division, two viable daughter cells replace the mother cell, and the distribution of the division times (i.e. the times from birth to division for each single cell) does not depend on the number of ancestral generations. This is expressed by the fact that the above equation is autonomous; that is the time does not explicitly appear on the right hand side of the differential equation. Given constraints (2)-(5), the $(\dot{y}, y)$ phase-plane portrait of the initial value problem, (6), follows the pattern shown by the black dashed line in Figure 1 of the main text; it decreases monotonically as the population size grows.

The model given in (6) expresses the transition from the exponential to stationary phases, but not the lag phase. The right hand side of our equation needs to be modified by an adjustment factor which indicates the time elapsed from the inoculation. This function should be small close to the inoculation and should increase monotonically, converging to 1 as the process arrives at the exponential phase. This factor makes the system non-autonomous (the left-hand side, $\dot{y}$, depending explicitly on time too, rather than implicitly through y). Such a factor was introduced in reference [3] in the form of a Michaelis-Menten function, $\alpha(P)$, where the "bottle-neck" substance, P, was produced at a constant rate, lending $\alpha(t)$ the well-known logistic shape. The blue line in Figure 1 in the main text shows the desired pattern of the phase-plane portrait for the non-autonomous model, with lag phase included. Assuming $\alpha(0)$ is sufficiently small, the solution of the initial value problem (6) follows a sigmoid pattern for $y = \ln x$, where a relatively linear phase is both preceded and followed by a transition phase characterised by small specific growth rates.

2 Prescribing versus inferring the noise level, σ

When deciding whether to infer or fix the noise level, σ, in our analysis, it is important to investigate how much difference its value makes to our results. For this reason we performed a comparison using the large dataset of growth curves available at ComBase. The Bayesian analysis was run twice over the dataset, fitting every curve using the 4 parameter model of Baranyi and Roberts as described in the main text (Equations (1) and (2)). The first of these times, the noise level was inferred (allowing us to obtain the value $\sigma = \sigma_I$), whilst the second time σ was fixed at $\sigma_F = 0.5$. The evidence was then compared for the two cases. The results are shown in Figure S1. Unsurprisingly, it was found that if σ is chosen correctly, there is not much difference between the two cases. However, if σ is prescribed incorrectly, the Bayes factor shows a large difference in evidence compared to the case where σ is inferred; the evidence being strongly in favour of the latter. Therefore, a good general rule would be that if we are not sure of the value of the noise level, we are best to infer it.

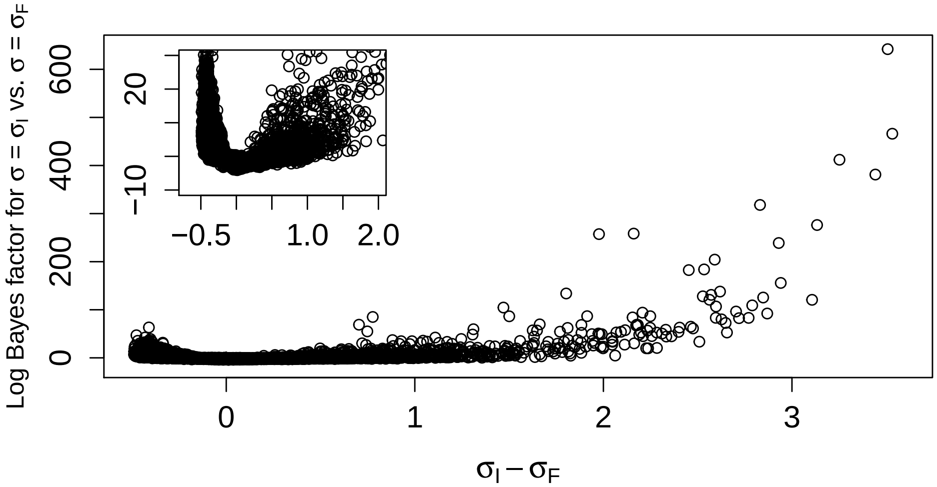

Figure S1: **If we are not confident in our estimate for the noise level then it should be inferred, since this can have a big impact on the results.** A large dataset comparison of the difference in evidence between the case where σ is inferred (with value σ_I) and prescribed at $\sigma_F = 0.5$. The inset shows a close up of the plot. Here we compare our results by plotting the log Bayes factor, $\ln \beta_{12}$. These results show that choosing to prescribe σ incorrectly can have a detrimental effect on the evidence, and therefore the results.

3 An explanation of the choice of control parameters used in the analysis

We investigated the best control parameters of our nested sampling code for accurate estimation of the evidence. For this, we used the 4 parameter model of Baranyi and Roberts as given in the main text (Equations (1) and (2)). In low-dimensional cases, the evidence can be approximately calculated by brute-force numerical integration. Three bacterial growth curves in the ComBase database were fitted using the 4 parameter model of Baranyi and Roberts and the evidence was evaluated on a fine grid with small step sizes for each of these. The output from 50 runs of nested sampling compare very well to the brute-force values for a range of prior sizes (Figure S2). On average nested sampling produces an estimate for the evidence that is accurate for practical purposes (Figure S3), where small differences in evidence suggests the data does not strongly discriminate between competing models. We note that there is variability in this stochastic algorithm due to differences in the random seed (Figure S4), but results are similar for enough samples, around 100. For the nested sampling algorithm a greater sampling density from the prior distribution will increase the chances of highly probable areas being explored. The current literature of nested sampling applications in biological problems use a large range of different prior sizes. For example, in the study of protein folding [4] a set of 20000 prior objects was used to provide a wide selection of conformations. At the other end of the scale it has been shown that maintaining a set of 25 active points can produce accurate parameter mean and standard deviations that are relatively insensitive to the prior size [5]. In our testing phase we found that 100 prior samples were often sufficient for the estimation of bacterial growth curve parameters and evidence.

There is no rigorous termination criterion to suggest when we have accumulated the bulk of the evidence [6], however one plausible criterion is that termination is decided by approximating the remaining evidence that can be accumulated from the posterior. This amount can be estimated as $\Delta\mathcal{Z}_i = \mathcal{L}_{\max} X_i$,

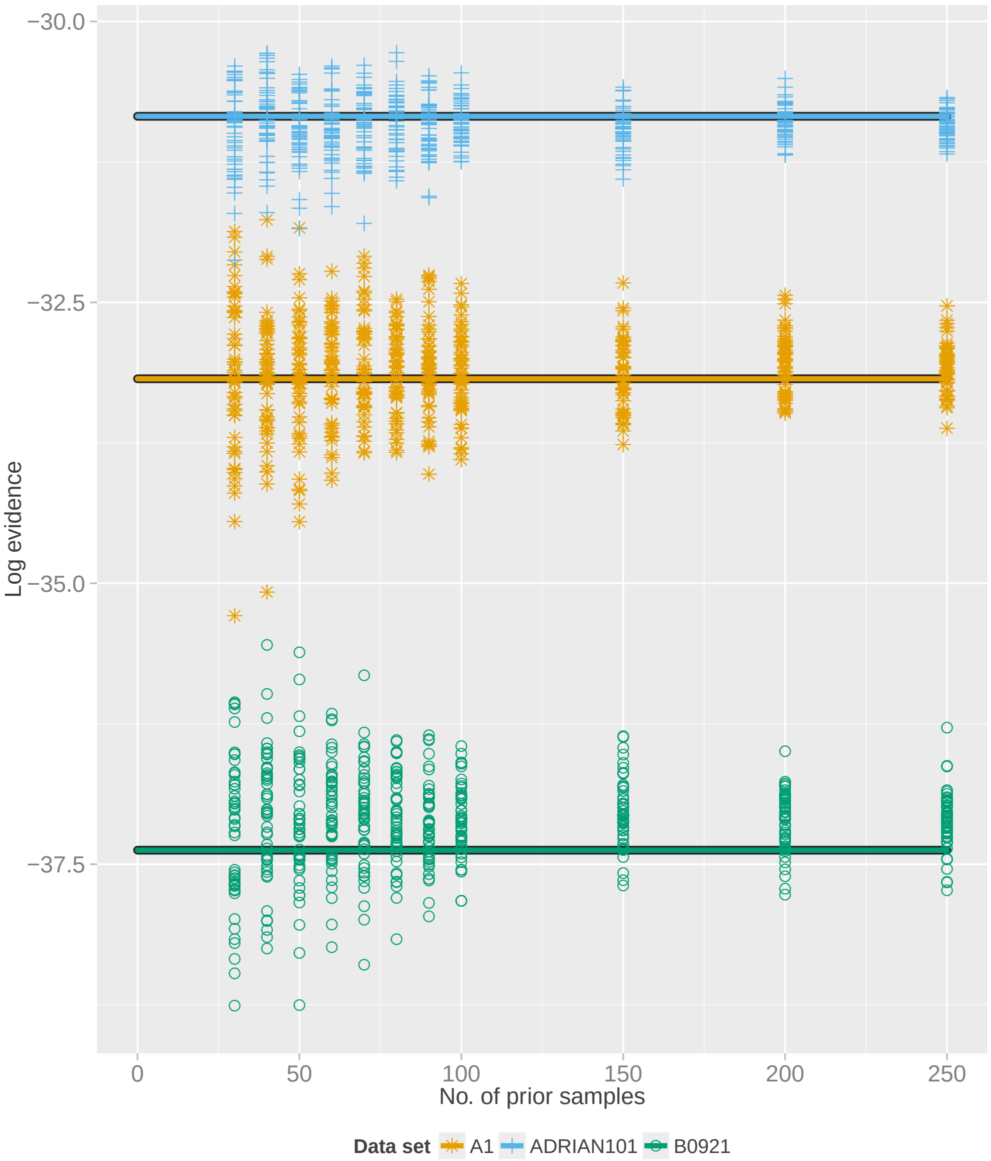

Figure S2: **Nested sampling accurately calculates the evidence for bacterial growth curve data.** The log evidence for 50 repeats of the analysis (using the 4 parameter model of Baranyi and Roberts) as a function of the number of prior samples is presented along with an approximation of the true evidence using brute-force numerical integration (lines) for each dataset. Three ComBase curves were used: A_1 (gold asterisks), B092_1 (green circles) and ADRIAN_101 (blue crosses).

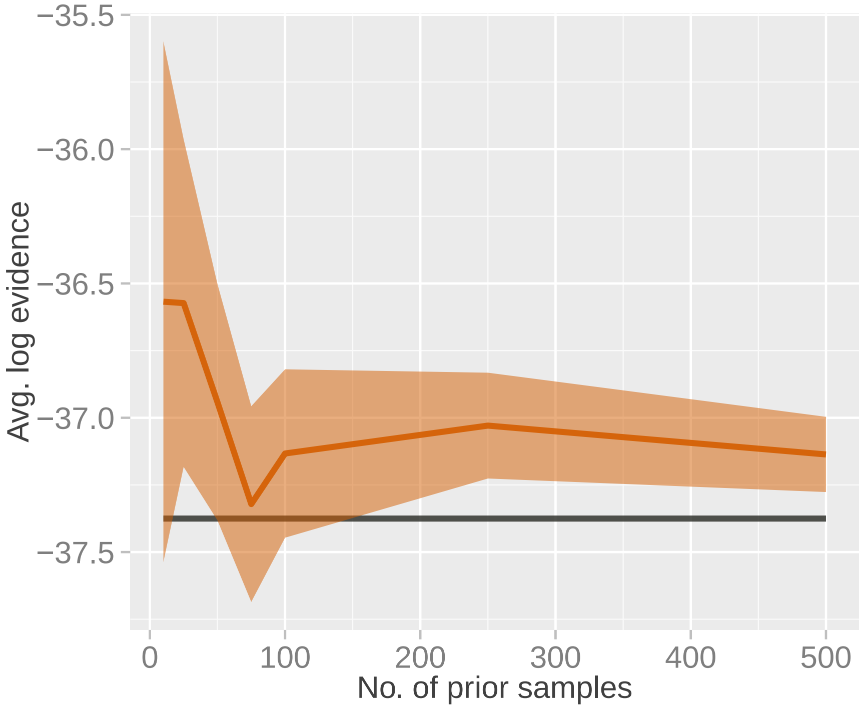

Figure S3: **A larger number of prior samples increases the accuracy and decreases the variance of the evidence, but the gain is not large above around 100 points.** The average log evidence plotted against the number of prior samples from 10 runs of nested sampling is shown as a thick orange line. The ribbon represents the average of the associated standard deviation of each run. Notice how it tightens around the mean for more prior samples. The dark line is the approximate value from brute-force numerical integration. Noting the scale of the y-axis, we see that it is not worth the expense of the extra computation time that is required for only slightly more accurate evidence calculation at larger numbers of prior samples.

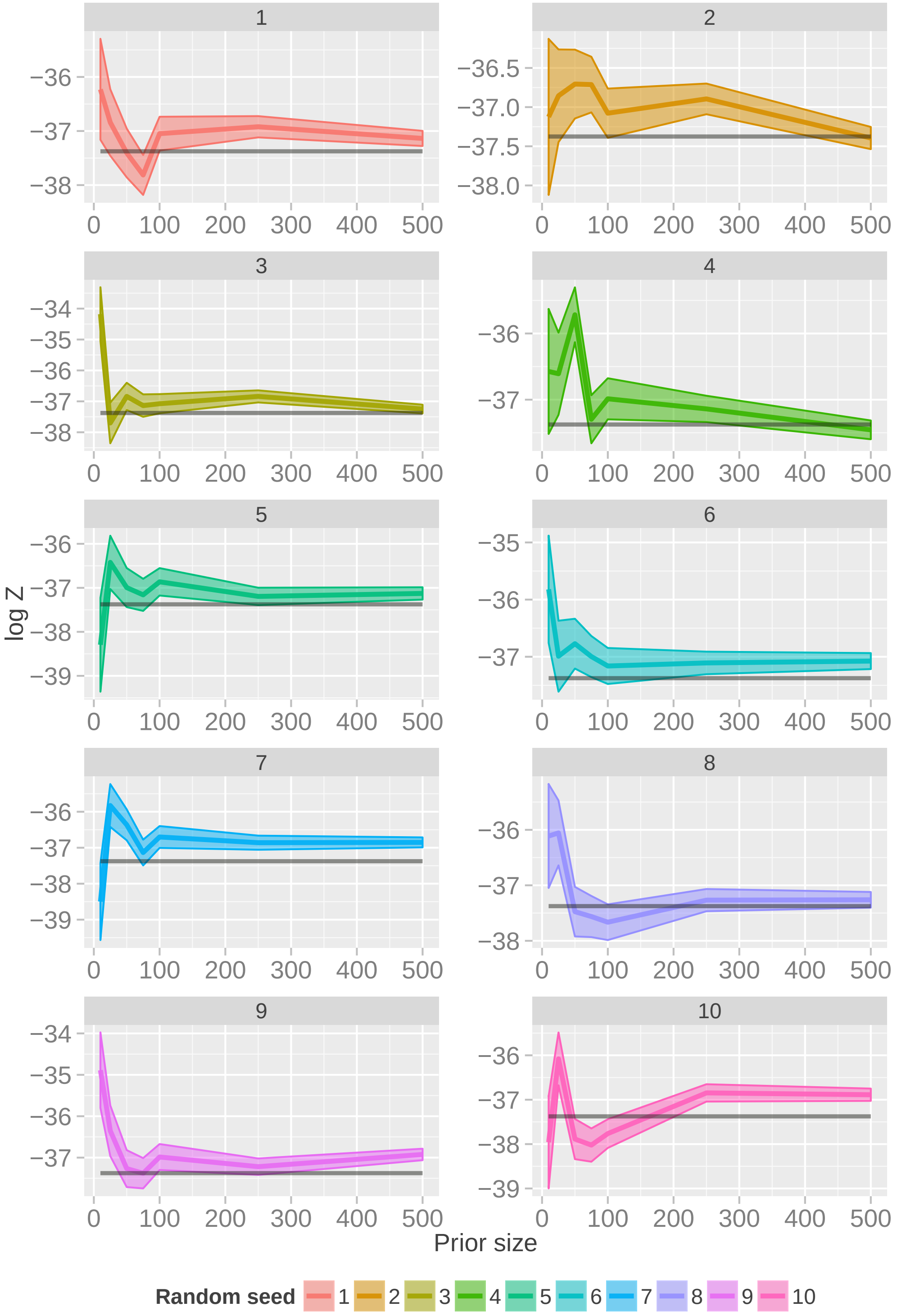

Figure S4: **Different random seeds introduce variation between runs of nested sampling.** Each plot shows the evidence from running the nested sampling with a different random seed as a function of prior size. This shows the variation one might expect in applications of the algorithm. Thick coloured lines show calculated log evidence and ribbons the associated standard deviation. The dark line is the approximate value from brute-force numerical integration.

where $\mathcal{L}_{\max}$ is the maximum likelihood value of the active set and X_i is the remaining prior volume [7, 8]. Using a selection of tolerance levels, it was found that one particular choice did not affect the accuracy of the evidence calculation (see Figure S5, for which each panel looks the same). To be safe, a tolerance of 0.1 was chosen in the log-evidence calculation. The number of posterior samples dictated by this criteria was checked against test cases (inferring bacterial curve parameters) with increasing numbers of posterior samples, and was found to be consistent.

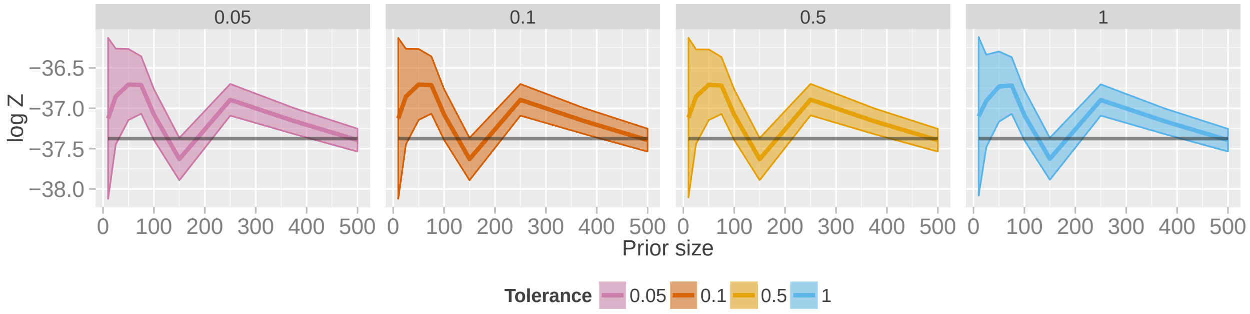

Figure S5: **Different termination tolerances have little effect on the calculated evidence.** Each plot shows the evidence from running the nested sampling with a different tolerance as a function of prior size. When comparing a selection of termination tolerances (0.05, 0.1, 0.5, 1) the similarity between plots demonstrates the tiny effect that tolerance has on the log evidence. Thick coloured lines show calculated log evidence and ribbons the associated standard deviation. The dark line is the approximate value from brute-force numerical integration.

4 Model comparison using the *Bayesfit* function

4.1 A set of models for bacterial growth

Although in the main text we focus on the 4 parameter model of Baranyi and Roberts (Equations (1) and (2)), we may instead consider a fairly all-encompassing set of growth models, which may be described as subsets of the 6 parameter model of Baranyi and Roberts [9, 3]. If we let the bacterial concentration at time t be given by $x(t)$, then this model is given by

$$y(t) = \ln x(t) = y_0 + \mu_{\max} A(t) - \frac{1}{m} \ln\left(1 + \frac{e^{m\mu_{\max} A(t)} - 1}{e^{m(y_{\max} - y_0)}}\right), \tag{7}$$

$$A(t) = t - \lambda + \frac{\ln\left(1 - e^{-\nu t} + e^{-\nu(t-\lambda)}\right)}{\nu}, \tag{8}$$

where $y_0 = \ln x(0)$ and $y_{\max} = \ln x_{\max}$, for $x_{\max}$ the maximum bacterial concentration. Here, $\mu_{\max}$ denotes the maximum specific growth rate in $(t, \ln x)$-space and m controls the curvature of the growth curve during the transition to the stationary phase. This model includes an adjustment function, which takes into account some intracellular substance that must be built up at some rate, ν, during the lag phase in order for bacterial growth to occur and which is assumed to follow Michaelis-Menten kinetics. This function introduces a lag phase of length λ. We note that although that the bacterial concentration is transformed to the $\ln x$ scale for use in the model, all results are transformed back afterwards to the $\log_{10} x$ scale.

Simpler models that include the adjustment function may be obtained given certain assumptions. In particular, if we assume that bacterial growth occurs at the same rate as growth of the rate-limiting substance so that $\mu_{\max} = \nu$, as well as setting $m = 1$ (a common choice for a transition to stationary phase that is not too sharp) and $h_0 = \lambda\mu_{\max}$ (so that the lag time is defined by the point at which the tangent to the

growth rate crosses the y axis divided by the growth rate), then the 4 parameter model is obtained, with parameter set $\{y_0, y_{\max}, \mu_{\max}, h_0\}$. Furthermore, if we take $m = 0$ instead of 1, then the stationary phase is lost, resulting in a 3 parameter model with parameter set $\{y_0, \mu_{\max}, h_0\}$.

We also consider a couple of classical growth models. These may be obtained by first replacing $A(t)$ by t, so that we lose the lag phase. Then, in the case of letting $m = 1$, the logistic model is obtained (a classical sigmoidal model) with parameter set $\{y_0, y_{\max}, \mu_{\max}\}$, whilst the choice $m = 0$ results in a linear model with parameter set $\{y_0, \mu_{\max}\}$. This subsetting of the 6 parameter model is summarised in Figure S6.

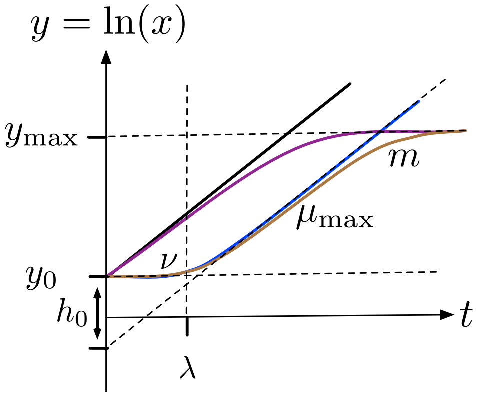

Model	Curve shape	Parameters
linear		y_0, $\mu_{\max}$
logistic		y_0, $y_{\max}$, $\mu_{\max}$
3 parameter model of Baranyi and Roberts		y_0, $\mu_{\max}$, h_0
4 parameter model of Baranyi and Roberts		y_0, $y_{\max}$, $\mu_{\max}$, h_0
6 parameter model of Baranyi and Roberts		y_0, $y_{\max}$, $\mu_{\max}$, λ, ν, m

Figure S6: **Simpler growth models can be considered as subsets of the 6 parameter model of Baranyi and Roberts [9, 3].** A summary of the bacterial growth models studied. The table gives a colour key for the curve shape for each model (the 4 and 6 parameter models share the same curve shape) and the parameters used in each model. Here $y_0 = \ln x_0$, where x_0 is the initial bacterial concentration and $y_{\max} = \ln x_{\max}$ where $x_{\max}$ is the maximum of the bacterial concentration. In addition, $\mu_{\max}$ is the maximum specific growth rate, λ is the lag time, $h_0 = \mu_{\max}\lambda$, and ν and m control the curvatures from the lag to exponential and exponential to stationary phases respectively.

4.2 Model comparison for comparing bacterial growth models

There are a number of software solutions available to implement the various growth models (as well as microbial survival or inactivation curves), but these offer only limited advice on model choice (usually through visual comparison of fitted curves combined with a statistical evaluation such as the coefficient of determination and goodness of fit). A summary of currently available resources is given in Table S1, including various tools available for the purposes of risk assessment.

As mentioned in the main text, by making use of the model comparison framework described in the methods section we may also compare bacterial growth models. We therefore take the opportunity to compare the above growth models over a large dataset. Since every extra parameter must be justified by the data [29], we must be careful not to inadvertently favour a more complicated model than necessary as this will lead to indeterminate parameters. A number of papers have used various statistical tests to compare

Table S1: Summary of resources available to model microbial growth and survival.

Name and reference	Microbial resources
ComBase [10]	Database of responses, predictive growth models
DMFit [11]	Predictive growth model
BGFit [12]	Dataset management, predictive growth models
BacSim [13]	Predictive growth model
grofit [14]	R package, dose-response curve modelling
cellGrowth [15]	R package, parametric and non-parametric fitting
microHibro [16]	Growth models in vegetable and meat products
Pathogen Modelling Program [17]	Growth models in vegetable and meat products
FISHMAP [18]	Growth models in fish products
Food Spoilage & Safety Predictor [19]	Growth models in food products
MLA Index Calculator [20, 21]	Log growth calculation in meat products
Shelf Stability Predictor [22]	Growth models in ready-to-eat meals
Salmonella Predictions [23, 24]	Excel add-in, response to fluctuating conditions
E. coli SafeFerment [25]	Excel add-in, response to fluctuating conditions
GInaFiT [26]	Excel add-in, predictive survival models
E. coli Inactivation Model [27]	Predictive survival models
Sym'previus [28]	Database of responses, predictive growth/survival models

models [30, 31, 32]. These papers have indicated that the most appropriate model may vary depending on the data, but that for minimal datasets, a simpler model may often be preferred over one with more parameters.

As discussed above, in the literature there has been a tendency to set $\mu_{\max} = \nu$ and $m = 1$ [9, 3, 33]. However, recent work using *Escherichia coli* suggests that in arabinose, $\mu_{\max} > \nu$ [34]. Using the 6 parameter model of Baranyi and Roberts, it is found that over a large dataset the means of the parameter samples are in agreement with $\mu_{\max} \neq \nu$ (see Figure S7). It is also found that typically $m \neq 1$. A further assumption

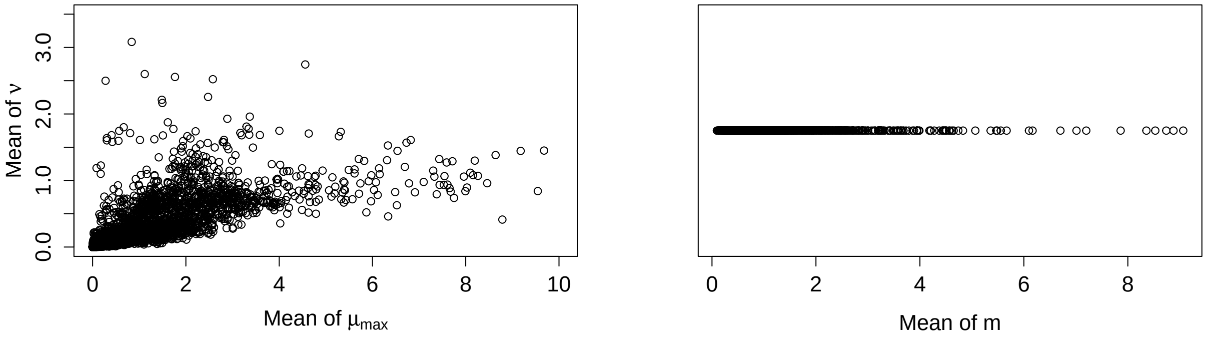

(a) (b)

Figure S7: **Contrary to the assumptions often made in growth models, $\mu_{\max} \neq \nu$, $m \neq 1$ over a large dataset.** Using the 6 parameter model of Baranyi and Roberts and inferring the noise level, σ, we plot the mean values of the parameters $\mu_{\max}$, ν and m over the dataset. We find that contrary to the simplifying assumptions made in several growth curve models, $\mu_{\max}$ is not always equal to ν and m is not always equal to 1.

(also discussed above) is to let $h_0 = \mu_{\max}\lambda$. In our analysis we find that as a direct result of this (and the assumption that $\mu_{\max} = \nu$), $\mu_{\max}$ and h_0 are more highly (positively) correlated in the 4 parameter model of Baranyi and Roberts than their counterparts, $\mu_{\max}$ and λ, in the 6 parameter model (in which λ and ν are also found to be significantly negatively correlated). This can be seen, for example, in Figure S8.

Despite these apparent differences between models, calculation of the evidence when fitting the various models to each single growth curve over a large dataset suggests that it is far from clear that one model fits better than another. Figure S9 shows the results of calculating the preferred model as a function of the number of data-points in a given curve. Here Bayes' factor is used together with the principle of Occam's razor, so that a more complicated model (with more parameters) is preferred only if there is substantial evidence for it over a simpler model. We also note that there are less data curves in the dataset as we tend towards large and small numbers of data points, explaining the bell-shaped pattern that can be seen in these plots. There are found to be some general rules in play. For instance, for just two or possibly three data points the most often preferred model is the simpler logistic model. Once there are more than two or three data points, the 6 parameter model is the most often favoured, followed by the 4 parameter model. However, the best model is not always obvious and certainly there is no clear rule as to which growth model is the most appropriate for a given set of data. Therefore, in agreement with the work of reference [30], we would recommend that a new model comparison analysis be undertaken for each new set of data, in order to find the optimum fit.

On the other hand, we must be careful not to be misled by statistical methods while comparing growth models and certainly should not blindly apply them without forethought. Often the knowledge and experience of the scientist may bring circumstantial evidence to the problem. For example, if the experiment is run over

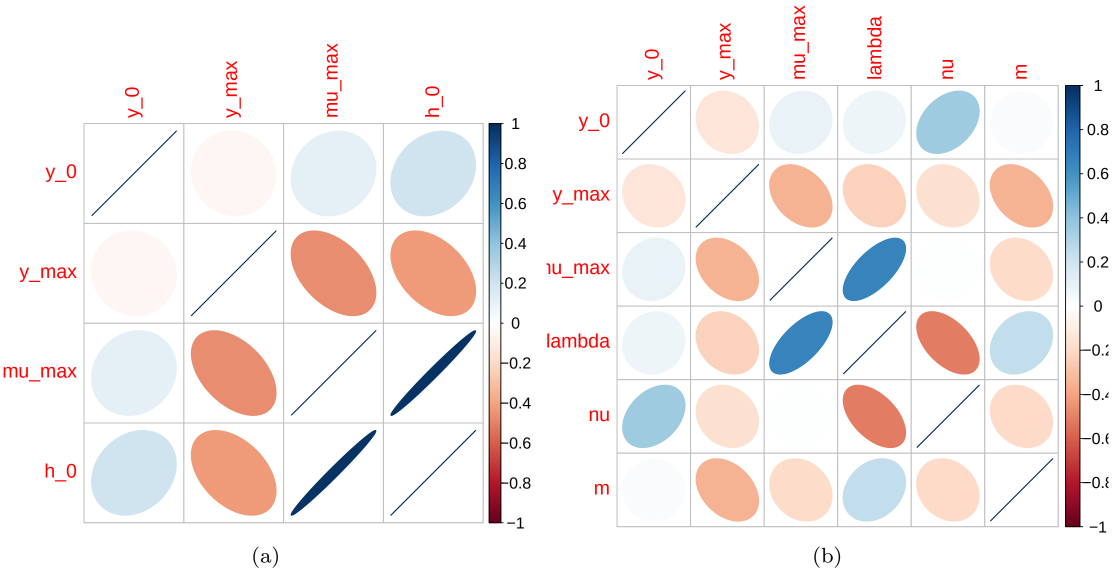

Figure S8: **The growth rate $\mu_{\max}$ is highly correlated with h_0.** A typical example (using curve A_1 from the ComBase database and prescribing the noise level, σ, at $\sigma_F = 0.5$) of correlation matrices for the (a) 4 and (b) 6 parameter models of Baranyi and Roberts. Here the orientation of ellipses represents the sign of the correlation. The correlation between $\mu_{\max}$ and h_0 in the 4 parameter model is stronger than the correlation between $\mu_{\max}$ and λ in the 6 parameter model.

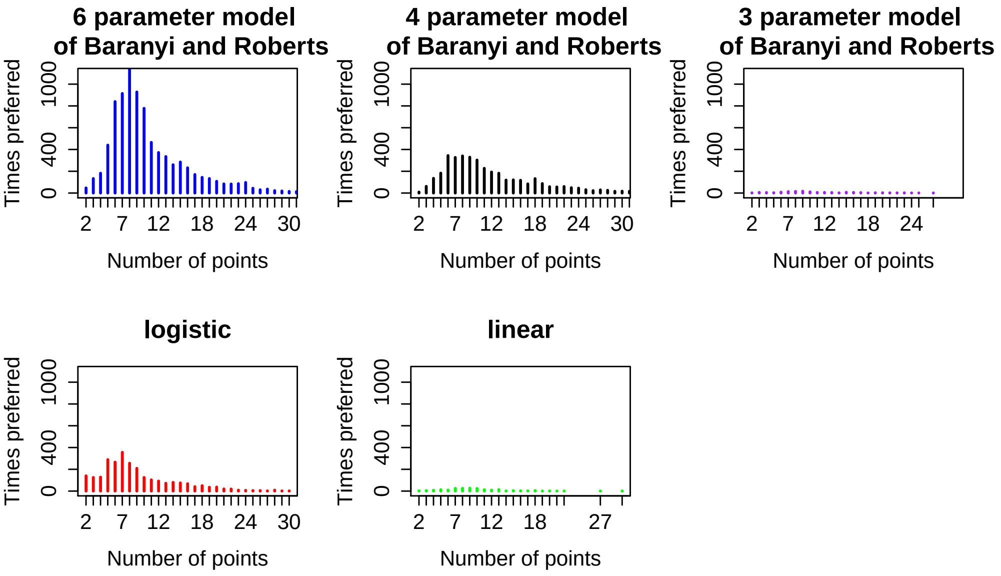

Figure S9: **There is no single preferred growth model, but the number of data-points plays a part.** The number of times each model is the most preferred over a large dataset as a function of the number of data points (where we note that there are less data curves in the dataset as we tend towards large and small numbers of data points). A model with more parameters is preferred only if there is substantial evidence for it over a model with fewer parameters. The noise level, σ, is inferred.

a long time, the bacteria in question may be growing under stress conditions and the observed curve may actually be a combination of both growing and dying subpopulations. This sort of knowledge can only be added by the experimentalist and confirmed via experience during other similar experiments. Therefore we suggest that this kind of analysis be used as a complementary tool, together with the user's own experience, to ultimately determine the best model to be used in each situation and for each application.

5 Supplementary figures

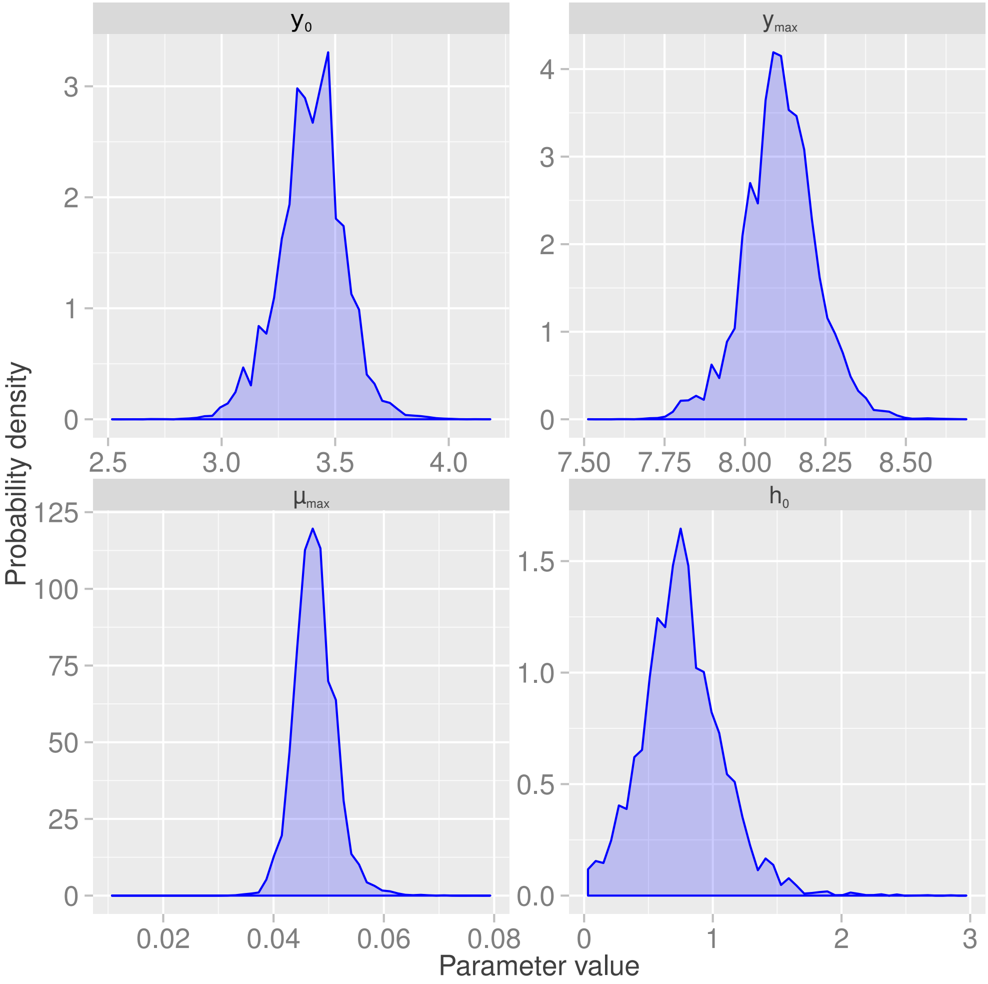

Figure S10: **The sampled posterior pdf is approximately unimodal.** A typical example of the marginal distributions for all of the parameters in the 4 parameter model of Baranyi and Roberts, obtained by sampling the posterior distributions. Here we use curve A_100 in the ComBase database and the noise level, σ, is inferred.

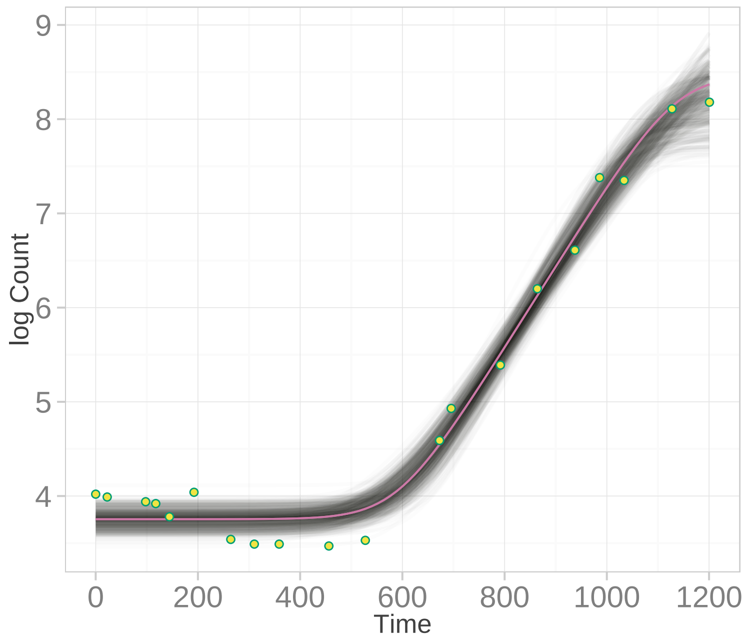

Figure S11: **The mean parameter values capture the posterior samples.** We performed nested sampling on data points from curve A_1 (yellow circles) from the ComBase database using the 4 parameter model of Baranyi and Roberts and with the noise in the error model prescribed at $\sigma = 0.3$. Staircase sampling was used to generate equally-weighted posterior parameter samples, which allows us to plot posterior trajectories. Grey-scale curves with darker colours indicate more posterior samples follow those trajectories. Fitting the model with the mean parameter values (pink) captures the posterior very well; the greatest number of posterior samples align with this curve, and there is a similar pattern for all samples.

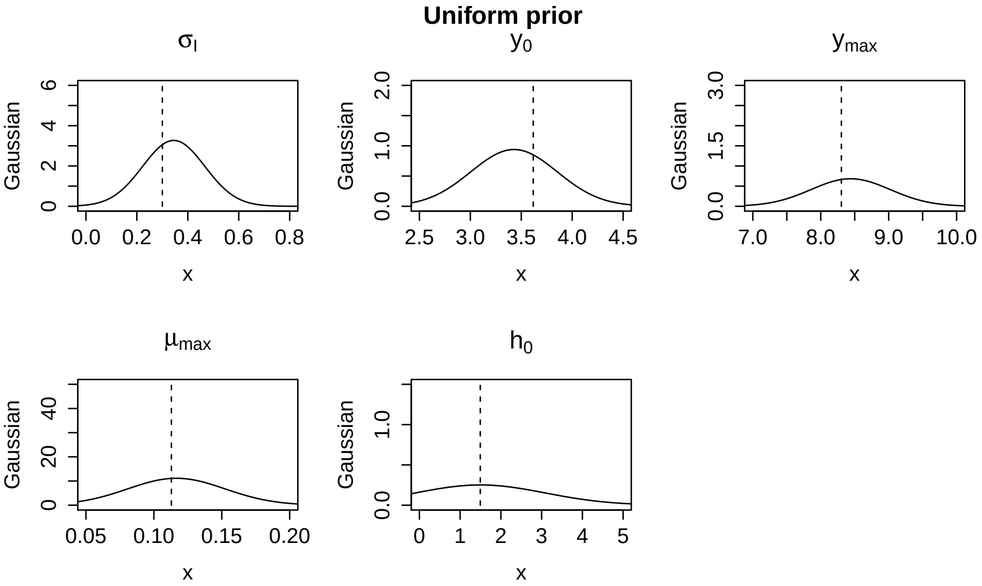

Figure S12: **The mean parameter values are in agreement with the true values, and we are also able to infer the noise level, σ.** An example of plotting Gaussian distributions using the mean and standard deviations of parameters for the 4 parameter model of Baranyi and Roberts when fitting a (known) test curve using Bayesian analysis. Here the noise level, σ, inferred (with value σ_I). The true values are shown by the vertical dashed lines.

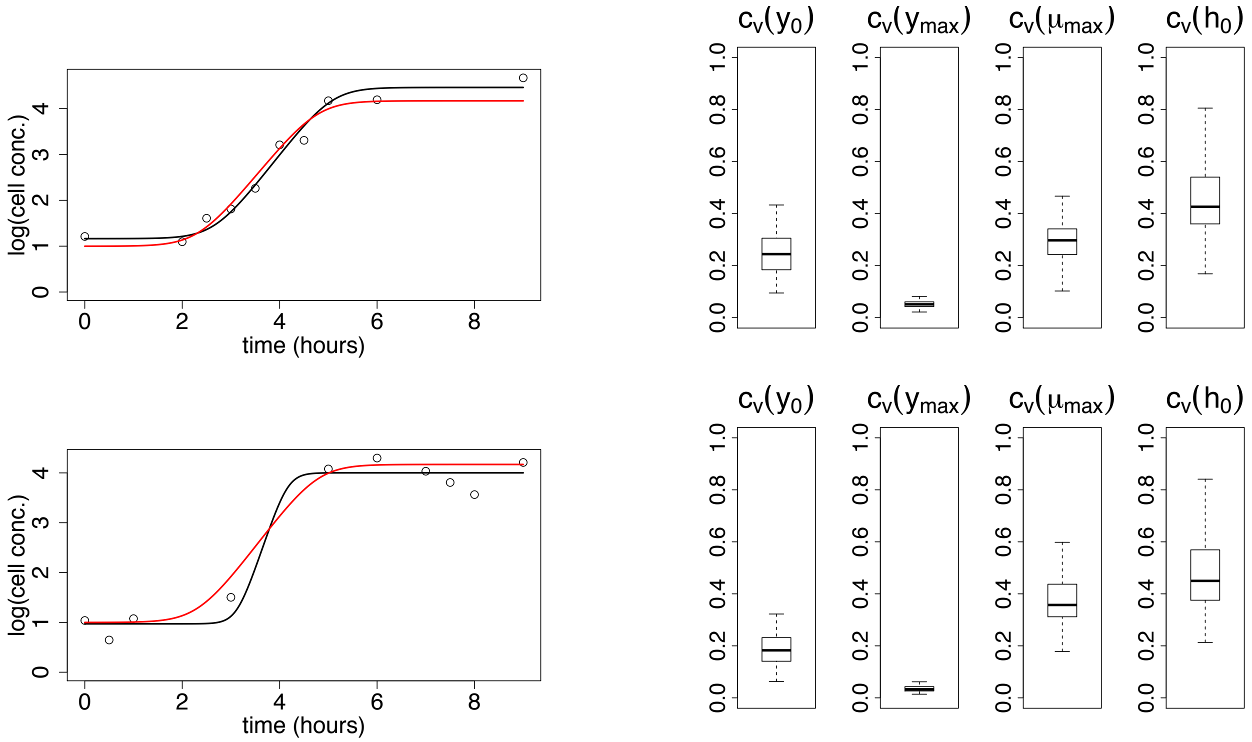

Figure S13: **The accuracy of growth rate estimation depends on the data quality.** The results from the Bayesian analysis when generating 50 curves, each with 10 data points and fitting using the 4 parameter model of Baranyi and Roberts. Examples are shown in the left panels, where circles represent the data points, the red line shows the true solution and the black line shows the solution resulting from the analysis. The coefficient of variation (c_v = standard deviation/mean) is computed for all 4 parameters for each curve and the overall results are given as box plots (right panels). The top two panels show the results when data is biased towards the growth region, whilst the bottom two show the results when data is biased against this region. Bayesian analysis captures the difference in quality between the two cases and this is reflected in the difference in the coefficient of variation for the growth rate, $\mu_{\max}$.

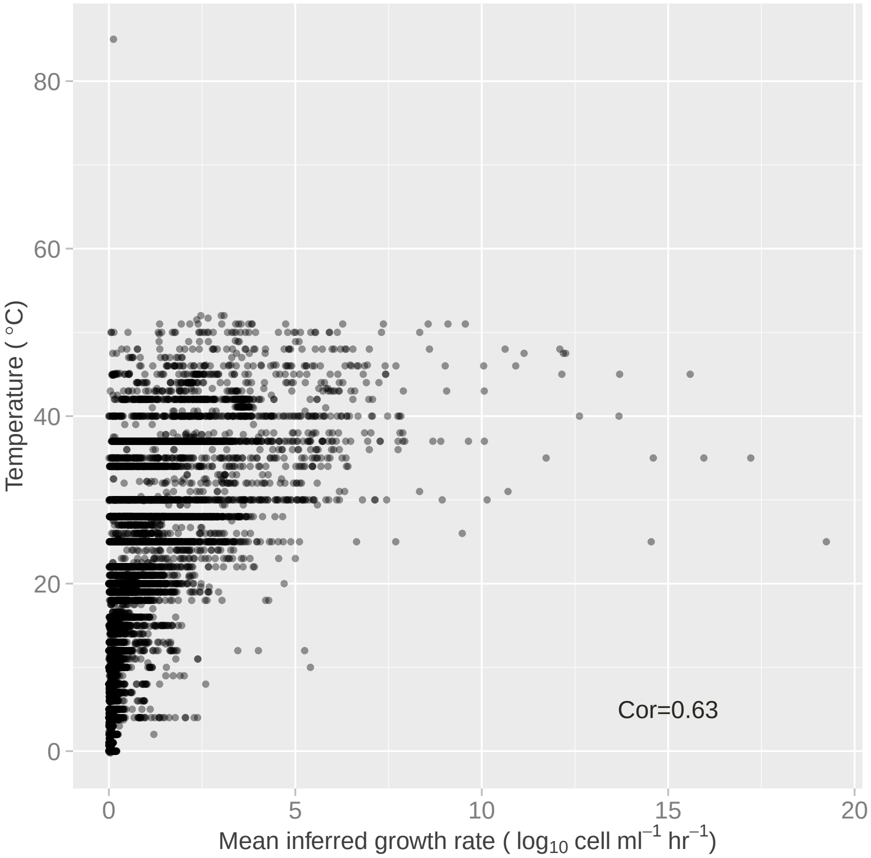

Figure S14: **There is a general correlation between temperature and bacterial growth rate.** Fitting all of the growth curves available at ComBase using the 4 parameter model of Baranyi and Roberts, we find an overall correlation of 0.63 between the temperature of each experiment and our inferred growth rate for that experiment.

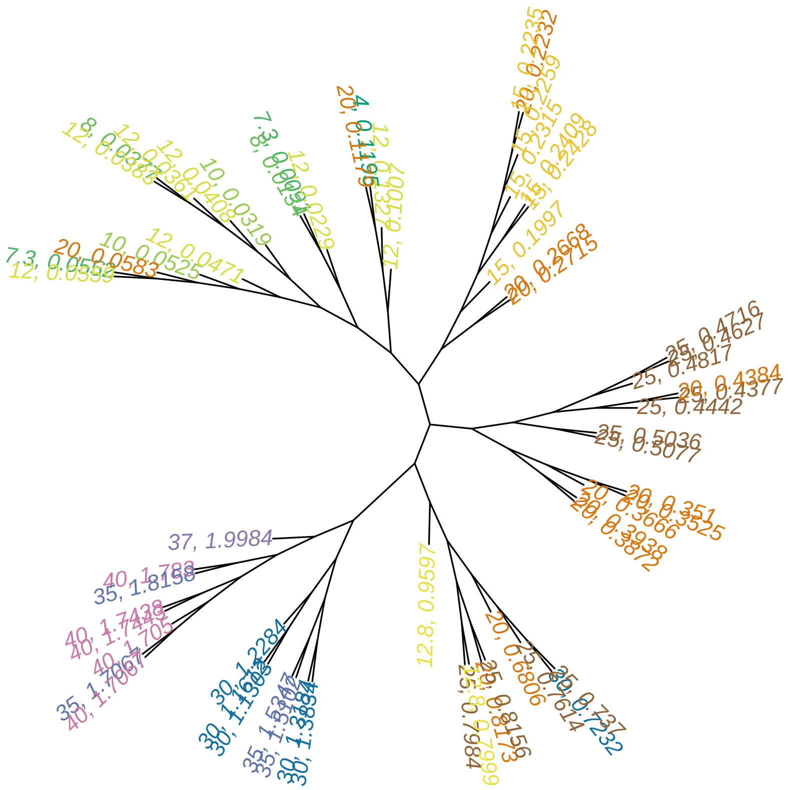

Figure S15: **Results may be clustered by estimated growth rate instead of temperature.** Growth curves for the organism *Salmonella spp.* at pH values of between 5 and 5.1 were clustered by their inferred growth rates instead of temperature and the coloured by the temperature. For each cluster entry, the first number is the temperature and the second is the growth rate. Again clusters with similar estimated growth rates are found to grow in similar temperatures (high temperature is purple, low temperature is green).

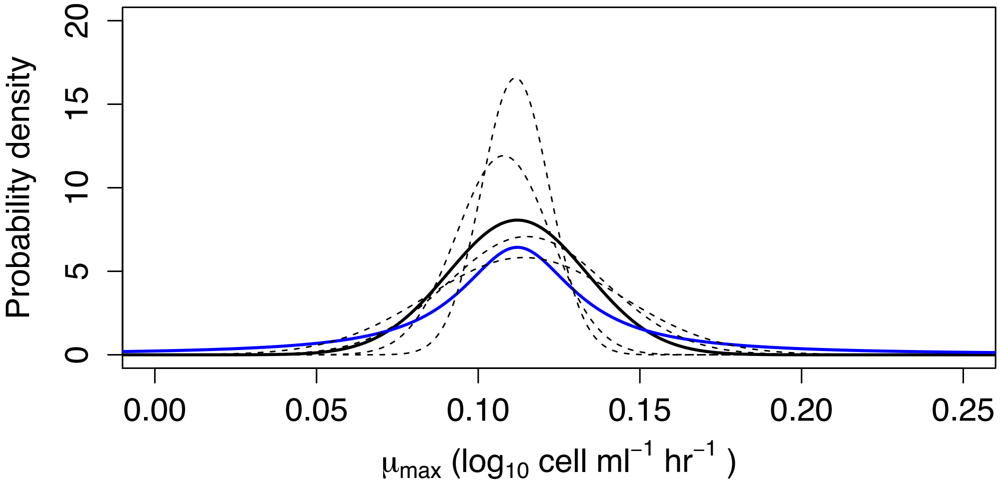

Figure S16: **Clusters of curves inform the prior.** A cluster of four curves is illustrated by plotting a Gaussian distribution using the mean and variance of each curve (dashed lines). The means and variances are combined using Equations (13) and (14) of the main text to give an overall mean and variance which is used to build both a Gaussian (solid black line) and Cauchy (solid blue line) prior.

References

[1] Turner, M.E., Bradley, E.L., Kirk, K.A., Pruitt, K.M.: A theory of growth. Math Biosci **29**(3-4), 367–373 (1976)

[2] Vance, R.R., Coddington, E.A.: A nonautonomous model of population growth. J Math Bio **27**, 491–506 (1989)

[3] Baranyi, J., Roberts, T.: A dynamic approach to predicting bacterial growth in food. Int J Food Microbiol **23**, 277–294 (1994)

[4] Burkoff, N., Várnai, C., Wells, S., Wild, D.: Exploring the energy landscapes of protein folding simulations with Bayesian computation. Biophys J **102**(4), 878–86 (2012)

[5] Aitken, S., Akman, O.: Nested sampling for parameter inference in systems biology: application to an exemplar circadian model. BMC Syst Biol **7**(1), 72 (2013)

[6] Sivia, D.S., Skilling, J.: Data Analysis: A Bayesian Tutorial. Oxford Science Publications. Oxford University Press, Oxford, UK (2006)

[7] Feroz, F., Hobson, M., Bridges, M.: MULTINEST: an efficient and robust Bayesian inference tool for cosmology and particle physics. Mon Not R Astron Soc **398**(4), 1601–1614 (2009)

[8] Feroz, F., Hobson, M., Cameron, E., Pettitt, A.: Importance nested sampling and the MultiNest algorithm. arXiv:1306.2144 (2013)

[9] Baranyi, J., Roberts, T., McClure, P.: A non-autonomous differential equation to model bacterial growth. Food Microbiol **10**, 43–59 (1993)

[10] ComBase. http://www.combase.cc/. Accessed 9 Feb 2015 (2013)

[11] DMFit. http://www.ifr.ac.uk/safety/dmfit/. Accessed 9 Feb 2015 (1994)

[12] Veríssimo, A., Paixão, L., Neves, A., Vinga, S.: BGFit: management and automated fitting of biological growth curves. BMC bioinformatics **14**, 283 (2013)

[13] Kreft, J., Booth, G., Wimpenny, J.: BacSim, a simulator for individual-based modelling of bacterial colony growth. Microbiology **144**(12), 3275–3287 (1998)

[14] Kahm, M., Hasenbrink, G., Lichtenberg-Fraté, H., Ludwig, J., Kschischo, M.: grofit : Fitting Biological Growth Curves with R. J Stat Softw **33**(7), 1–21 (2010)

[15] Gagneur J and Neudecker A: cellGrowth: Fitting Cell Population Growth Models. (2012). R package version 1.8.0.

[16] microHibro. http://www.microhibro.com. Accessed 10 Feb 2015 (2011)

[17] Pathogen Modelling Program. http://pmp.errc.ars.usda.gov/PMPOnline.aspx. Accessed 10 Feb 2015 (2005)

[18] FISHMAP. http://www.azti.es/downloads/fishmap/. Accessed 9 Feb 2015 (2013)

[19] Food Spoilage and Safety Predictor. http://sssp.dtuaqua.dk. Accessed 10 Feb 2015 (2014)

[20] Ross, T., Ratkowsky, D., Mellefont, L., McMeekin, T.: Modelling the effects of temperature, water activity, pH and lactic acid concentration on the growth rate of Escherichia coli. Int J Food Microbiol **82**(1), 33–43 (2003)

[21] Mellefont, L., McMeekin, T., Ross, T.: Performance evaluation of a model describing the effects of temperature, water activity, pH and lactic acid concentration on the growth of *Escherichia coli*. Int J Food Microbiol **82**(1), 45–58 (2003)

[22] Shelf Stability Predictor. http://meathaccp.wisc.edu/ST_calc.html. Accessed 10 Feb 2015 (2009)

[23] Pin, C., Avendaño-Perez, G., Cosciani-Cunico, E., Gómez, N., Gounadakic, A., Nychas, G.-J., Skandamis, P., Barker, G.: Modelling *Salmonella* concentration throughout the pork supply chain by considering growth and survival in fluctuating conditions of temperature, pH and a(w). Int J Food Microbiol **145**, 96–102 (2011)

[24] Quinto, E., Arinder, P., Axelsson, L., Heir, E., Holck, A., Lindqvist, R., Lindblad, M., Lauzon, H., Marteinsson, V., Pin, C.: Predicting the concentration of verotoxin-producing *Escherichia coli* during processing and storage of fermented raw meat sausages. Appl Environ Microbiol **80**(9), 2715–27 (2014)

[25] *E. coli* SafeFerment. http://www.ifr.ac.uk/safety/EcoliSafeFerment. Accessed 10 Feb 2015 (2014)

[26] Geeraerd, A., Valdramidis, V., Van Impe, J.: GInaFiT, a freeware tool to assess non-log-linear microbial survivor curves. Int J Food Microbiol **102**(1), 95–105 (2005)

[27] Ross, T., Shadbolt, C., Meat and Livestock Australia: Predicting Escherichia Coli Inactivation in Uncooked Comminuted Fermented Meat Products. Meat & Livestock Australia, Armidale, Australia (2001)

[28] Sym'previus. www.symprevius.org. Accessed 10 Feb 2015 (2015)

[29] Hawkins, D.: The problem of overfitting. J Chem Inform Comput Sci **44**(1), 1–12 (2004)

[30] Zwietering, M., Jongenburger, I., Rombouts, F., van 't Riet, K.: Modeling of the bacterial growth curve. Appl Environ Microb **56**(6), 1875–1881 (1990)

[31] Buchanan, R., Whiting, R., Damert, W.: When is simple good enough: a comparison of the Gompertz, Baranyi, and three-phase linear models for fitting bacterial growth curves. Food Microbiol **14**(4), 313–326 (1997)

[32] Baty, F., Delignette-Muller, M.-L.: Estimating the bacterial lag time: which model, which precision? Int J Food Microbiol **91**(3), 261–77 (2004)

[33] Baranyi, J.: Modelling and parameter estimation of bacterial growth with distributed lag time. PhD thesis, Doctoral School of Informatics, University of Szeged, Hungary (2010)

[34] Madar, D., Dekel, E., Bren, A., Zimmer, A., Porat, Z., Alon, U.: Promoter activity dynamics in the lag phase of *Escherichia coli*. BMC Syst Biol **7**(1), 136 (2013)
